# MutRank: an R shiny web-application for exploratory targeted mutual rank-based coexpression analyses integrated with user-provided supporting information

**DOI:** 10.7717/peerj.10264

**Published:** 2020-11-09

**Authors:** Elly Poretsky, Alisa Huffaker

**Affiliations:** Division of Biology, University of California, San Diego, La Jolla, CA, USA

**Keywords:** Shiny, Transcriptomes, Coexpression analyses, Mutual rank, Gene function prediction, Functional association, Pathway discovery, Specialized metabolism, Customizable, Plant biology

## Abstract

The rapid assignment of genotypes to phenotypes has been a historically challenging process. The discovery of genes encoding biosynthetic pathway enzymes for defined plant specialized metabolites has been informed and accelerated by the detection of gene clusters. Unfortunately, biosynthetic pathway genes are commonly dispersed across chromosomes or reside in genes clusters that provide little predictive value. More reliably, transcript abundance of genes underlying biochemical pathways for plant specialized metabolites display significant coregulation. By rapidly identifying highly coexpressed transcripts, it is possible to efficiently narrow candidate genes encoding pathway enzymes and more easily predict both functions and functional associations. Mutual Rank (MR)-based coexpression analyses in plants accurately demonstrate functional associations for many specialized metabolic pathways; however, despite the clear predictive value of MR analyses, the application is uncommonly used to drive new pathway discoveries. Moreover, many coexpression databases aid in the prediction of both functional associations and gene functions, but lack customizability for refined hypothesis testing. To facilitate and speed flexible MR-based hypothesis testing, we developed MutRank, an R Shiny web-application for coexpression analyses. MutRank provides an intuitive graphical user interface with multiple customizable features that integrates user-provided data and supporting information suitable for personal computers. Tabular and graphical outputs facilitate the rapid analyses of both unbiased and user-defined coexpression results that accelerate gene function predictions. We highlight the recent utility of MR analyses for functional predictions and discoveries in defining two maize terpenoid antibiotic pathways. Beyond applications in biosynthetic pathway discovery, MutRank provides a simple, customizable and user-friendly interface to enable coexpression analyses relating to a breadth of plant biology inquiries. Data and code are available at GitHub: https://github.com/eporetsky/MutRank.

## Introduction

Visually-apparent biological complexity is greatly exceeded by the extreme diversity of specialized metabolites made by organisms for the mediation of essential biotic and abiotic interactions (Dixon, 2001; Gershenzon & Dudareva, 2007; Pichersky & Lewinsohn, 2011). In plants, the ability to identify and control the production of specialized metabolites has significant implications for human health and agriculture; however, efficient tools aiding in biosynthetic pathway discovery remain limited (Dixon, 2001; Moghe & Kruse, 2018). Clustering of plant specialized metabolism genes has historically been a useful, but not the sole, indicator of functional associations, and has accelerated the discovery of multiple specialized metabolite biosynthetic pathways (Frey, 1997; Osbourn, 2010; Boutanaev et al., 2015). For the discovery of non-clustered metabolic pathway genes, coexpression analyses have emerged as a powerful predictive tool. Genes in specialized metabolic pathways are often highly coregulated based on developmental, spatial, environmental and complex regulatory controls (Schmelz et al., 2014; Lacchini & Goossens, 2020). Genes that work together in functional specialized metabolic pathways are likely to require transcriptional coregulation and thus resulting patterns used to predict both functional associations and putative gene functions (Chae et al., 2014; Wisecaver et al., 2017). With increasingly affordable and accessible next generation sequencing technologies, new public and private custom large-scale transcriptomic datasets are routinely generated (Zhou et al., 2020). Studies in plants often generate hundreds and even thousands of transcriptomic samples from different genotypes, developmental stages, tissues and physiological conditions to understand traits of agronomic significance (Sekhon et al., 2011; Stelpflug et al., 2016; Kremling et al., 2018; Machado et al., 2020). Moreover, genomes and transcriptomes from thousands of plant species are expected to speed large-scale gene expression experiments in poorly understood models (Twyford, 2018; One Thousand Plant Transcriptomes Initiative, 2019). Public and lab-specific transcriptomic resources are far from static, instead they are continuously expanding and dynamic resources that require flexible tools for rapid and effective analyses.

Many databases and webtools, such as PLEXdb (Dash et al., 2012), Genevestigator (Hruz et al., 2008), PLANEX (Yim et al., 2013), CORNET (De Bodt et al., 2010, 2012), ATTED-II (Obayashi et al., 2018), COXPRESdb (Obayashi et al., 2012), RiceFREND (Sato et al., 2013), ePlant (Waese et al., 2017) and STRING (Szklarczyk et al., 2019) have been developed to facilitate gene coexpression analyses. Coexpression analyses in studies and databases often use the Pearson’s Correlation Coefficient (PCC) as a measure of coexpression. Mutual Rank (MR), the geometric mean of the ranked PCCs between a pair of genes, has been further proposed as an alternative measure of coexpression to PCC (Obayashi & Kinoshita, 2009). MR-based coexpression analyses provide better indication of functional associations and are more robust to inconsistencies caused by different microarray data processing methods compared to PCC-based coexpression analyses (Obayashi & Kinoshita, 2009). Collective findings have driven some coexpression databases to use MR as the primary measure of coexpression (Obayashi et al., 2012, 2018; Sato et al., 2013). When the MR-and PCC-based coexpression databases of multiple plant species from ATTED-II (Obayashi et al., 2018) were converted into coexpression networks and compared, the MR-based coexpression networks were more comparable than PCC-based coexpression networks across species, suggesting that MR-based coexpression networks accurately represent functional associations (Wisecaver et al., 2017). MR-based coexpression networks enabled the accurate prediction of clusters enriched for enzymes associated with validated plant specialized metabolic pathways (Wisecaver et al., 2017). Wisecaver et al. (2017) further demonstrate that MR analyses of transcripts are an improved and powerful tool for the functional prediction of unclustered biosynthetic pathway genes to serve as a springboard for hypothesis testing and validation.

While coexpression databases are useful, few enable flexible hypothesis testing and tool-based simplicity that integrates user-provided data and information. Data integration with coexpression results facilitates the meaningful interpretation of predicted functional associations and assignment of putative gene functions. For example, if a cytochrome P450 monoxygenase (CYP) is hypothesized to perform an oxidation step in a specific biosynthetic pathway, a user might ask “which of all possible *CYP* transcripts is most highly coexpressed with an established pathway gene?”. More simply stated, any number of user-defined questions of targeted interest can be precisely examined. For any co-regulated process studied, the identification of 2–3 top candidates from a large gene family can greatly narrow efforts required for defined hypothesis testing and iterative re-testing. Towards this goal, we developed an R Shiny web-application, termed MutRank, to facilitate user control over both targeted and non-targeted MR-based coexpression analyses for rapid hypothesis testing. Using the R Shiny framework, we designed a flexible coexpression analysis platform that combines R packages to easily analyze and integrate user-provided expression data and information. Shiny web-applications are also advantageous for generating highly customizable and user-friendly interfaces that can run on most personal computers. In addition to identifying highly coexpressed genes in any user-provided dataset, MutRank automatically integrates supporting information such as gene annotations, differential-expression data, predicted protein domains and assigned Gene Ontology terms to provide useful tabular and graphical outputs as foundation for empirical hypothesis testing. Confirmed through diverse approaches, targeted and untargeted MR-based coexpression tools were recently leveraged to narrow gene candidates and accurately predict enzymes within multiple maize antibiotic biosynthetic pathways (Ding et al., 2019, 2020). The goal of MutRank is to provide simple, customizable and readily accessible tools to speed research progress by using exploratory targeted coexpression analyses to predict gene functions and functional associations.

## Methods

### Software packages and example supporting information used

**Figure 1:**
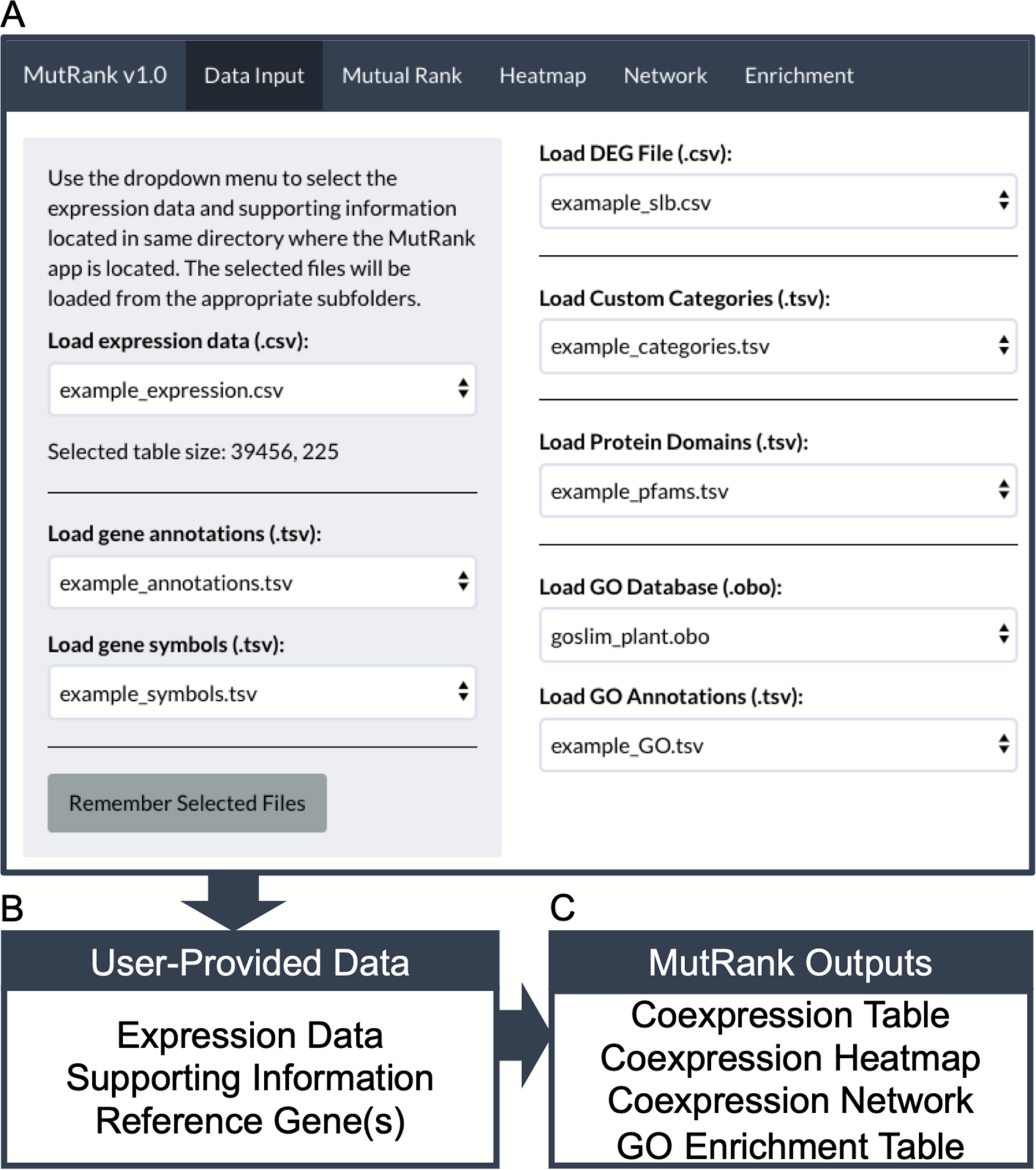
MutRank interface and workflow chart. (A) MutRank workflow starts at the Data Input tab at the top navigation bar that allows the selection of files to load and access different sections of MutRank. In the side panel users can select expression data files, gene descriptions and symbol annotations. In the main panel users can select additional supporting information which includes differential-expression data, custom categories, protein domains, and the Gene Ontology (GO) database along GO assignments. (B) With the user-provided expression data and integrated supporting information users can then select a single target reference gene or gene list to produce a (C) Mutual Rank-based coexpression table and to view the coexpression analysis results as a coexpression heatmap, coexpression network and a GO term enrichment table.

MutRank was developed as a web application using the Shiny R package (1.4.0.2) (Chang et al., 2020) that creates the user interface and manages navigation across the different application components (Fig. 1A). It requires R (3.4.0) and Java (Version 8 Update 261) to be installed by the user, and the following R packages will be automatically installed: shiny (1.4.0.2) (Chang et al., 2020), hypergea (1.3.6) (Boenn, 2018), ontologyIndex (2.5) (Greene, Richardson & Turro, 2017), reshape2 (1.4.3) (Wickham, 2007), RColorBrewer (1.1-2) (Neuwirth, 2014), data.table (1.12.8) (Dowle & Srinivasan, 2020), ggplot2 (3.3.0) (Wickham, 2016), visNetwork (2.0.9) (Almende, Thieurmel & Robert, 2019), igraph (1.2.4.2) (Csardi & Nepusz, 2005) and shinythemes (1.1.2) (Chang, 2018). To explain the features included in MutRank and to understand the required file structures we provide example expression data and supporting information. All the files used for examples are based on the *Zea mays* inbred B73 (RefGen_v3) genome annotation. The expression data is from the Expanded Maize Gene Expression Atlas (Stelpflug et al., 2016) (Fig. 1A; Table S1), gene annotations from the Phytozome database (Goodstein et al., 2012) (Fig. 1A; Table S2), and gene symbols from MaizeGDB (Portwood et al., 2019) (Fig. 1A; Table S3). Additional supporting information can be selected in the main panel (Fig. 1A). As an example of analyzing a custom dataset, differential expression data was obtained for maize stems 24 hours after treatment with a fungal pathogen, specifically Southern leaf blight (SLB; *Cochliobolus heterostrophus*) (Ding et al., 2019) (Table S4). The predicted Pfam protein domain annotations and GO term assignments are derived from the Phytozome database (Goodstein et al., 2012) (Tables S5 and S6). The GO-basic and Plant-GO-Slim ontologies are from the GO Consortium (Ashburner et al., 2000; The Gene Ontology Consortium, 2019). Lists of maize terpene synthases (TPS) (Ding et al., 2020), cytochrome P450s (CYP) (Ding et al., 2019) and Pfam protein domains associated with specialized metabolism (SM) (Wisecaver et al., 2017) were used as categories to assign to coexpressed genes (Table S7).

### Calculating mutual rank values

**Figure 2:**
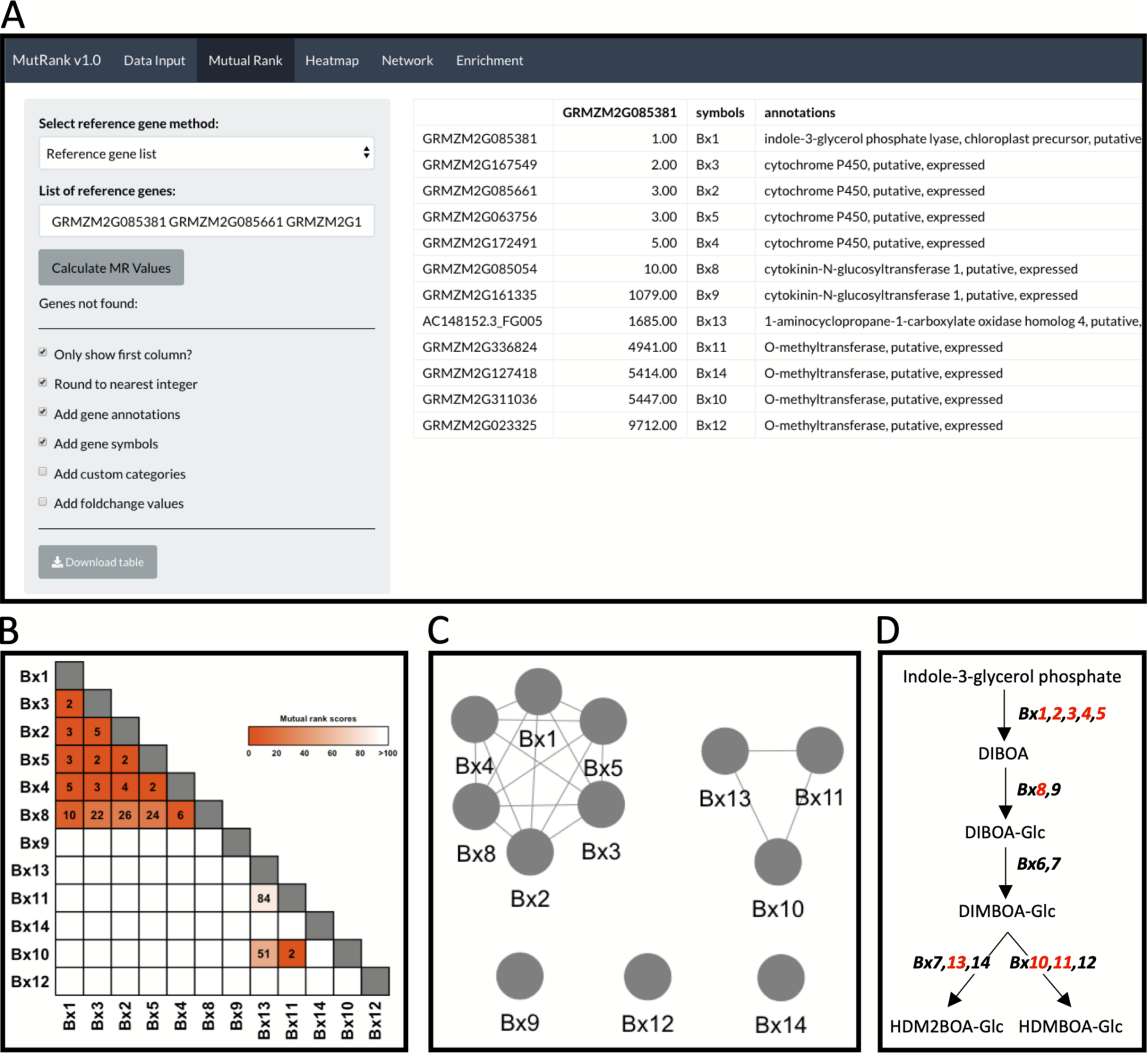
Example workflow 1: validation of MutRank using a characterized biosynthetic pathway. (A) In the Mutual Rank (MR) tab we used the reference gene list method with the characterized known enzymes in the benzoxazinoid (BX) biosynthetic pathway (*Bx1* to *Bx14*, note: *Bx6* and *Bx7* are absent from the example expression data) with default output, but excluding custom categories and fold-change values, to calculate the MR values and produce the MR coexpression table integrated with supporting information. The coexpression analysis results can be presented as a (B) coexpression heatmap and as a (C) coexpression network with an MR < 100 threshold for drawing edges between vertices showing two clusters of coexpressed genes. (D) Summarized diagram of the maize BX biosynthetic pathway with genes that were highly coexpressed designated in red.

MutRank was developed as a user-friendly tool to quickly identify the most highly coexpressed genes based on MR values for any reference gene and expression dataset. One of the limitations of MutRank is that it does not calculate all pair-wise MR values. Unlike coexpression databases that pre-calculate all pair-wise MR values (Obayashi et al., 2012, 2018; Sato et al., 2013), calculating all pair-wise MR values on the resources available on most personal computers is impractical. Instead, MutRank calculates all PCC values between the user-provided reference gene and all other genes to generate a limited list of genes with the highest PCC values (top 200 genes by default, maximum 1,000) for which it is feasible to calculate MR values. This practical trade-off between whole-genome and targeted coexpression analyses allows MutRank to rapidly complete calculations and to run on the resources of most personal computers. In addition to using a single reference gene, MutRank offers two additional methods for user-defined reference gene sets (Figs. 1B–2). The first method calculates the MR values between all genes in the reference gene set. The second method creates a novel compound reference gene from the average, sum, maximum or minimum expression values of the reference gene set. Using compound reference genes is important for capturing pan-genome patterns with key gene family members displaying highly variable expression across the analyzed germplasm (Ding et al., 2020).

### Integrating user-provided supporting information

As an exploratory targeted coexpression analysis tool, MutRank integrates user-provided supporting information with the identified list of coexpressed genes (Fig. 1B). Users can provide gene annotations and symbols as easy-to-read information connected to the identified list of coexpressed genes. Additional supporting information in the form of lists of differentially-expressed genes, predicted Pfam domains and assigned Gene Ontology (GO) terms can be integrated with the coexpressed genes. Users can also define custom categories made from lists of genes, Pfam domains or GO terms. The goal of assigning a gene in the MR-based coexpression results as belonging to any of the categories is to have a noticeable indication that the gene is either present in the gene list or is assigned at least one of the Pfam protein domains or GO terms.

### Tabular and graphical outputs for coexpression analyses

The primary output is provided in the form of an MR coexpression table (Fig. 1C). User-provided supporting information can be automatically integrated into the table in separate columns for each of the coexpressed genes. The results from the MR coexpression table are used as the basis for three additional informative outputs: heatmap, network graph and a GO enrichment table (Fig. 1C). The heatmap, generated using ggplot2 (Wickham, 2016), provides an overview of the distribution of MR values among the top coexpressed genes. The R igraph package (Csardi & Nepusz, 2005) is used to convert the coexpression table into a coexpression network and to annotate the gene vertices with user-provided data. The network graph visualization is produced with visNetwork package (Almende, Thieurmel & Robert, 2019) which allows the user to explore a dynamic network representation with supporting information. GO term enrichment is calculated using the hypergeometric test based on the GO database and all genes with MR values below a user-provided threshold (default MR < 100). The *P*-values are adjusted for false discovery rate and the results are presented in a separate table.

## Results

### Example workflow 1: integrating coexpression analyses of genes encoding a specialized metabolic pathway with supporting information

In maize and other important grain crops, benzoxazinoids (BXs) are a highly-studied class of nitrogen-containing specialized metabolites with critical roles in plant protection against both herbivores and pathogens (Frey, 1997; Meihls et al., 2013; Wouters et al., 2016). Genes underlying early steps in the maize BX biosynthetic pathway, namely *Bx1* to *Bx8*, are consitutively expressed in seedlings and drive the production of 2,4-dihydroxy-7-methoxy-1,4-benzoxazin-3-one glucoside (DIMBOA-Glc). A majority of these genes, *Bx1* to *Bx5* and *Bx8*, are located together on chromosome 4 and represent the first biosynthetic gene cluster ever described in plants (Frey, 1997; Dutartre, Hilliou & Feyereisen, 2012). In contrast to largely constitutive production, the late stage BX pathway, namely *Bx10* to *Bx14* and encoded enzymes, display stress-inducible regulation resulting in the conversion of DIMBOA substrates to 2-(2-hydroxy-4,7-dimethoxy-1,4-benzoxazin-3-one)-β-D-glucopyranose (HDMBOA-Glc) and 2-(2-hydroxy-4,7,8-trimethoxy-1,4-benzoxazin-3-one)-β-D-glucopyranose (HDM_2_BOA-Glc), which upon aglycone liberation (HDMBOA and HDM_2_BOA) result in highly unstable bioactive molecules (Maresh, Zhang & Lynn, 2006; Meihls et al., 2013; Wouters et al., 2016). While displaying complex regulation of early- and late-stage *Bx* genes influenced by development and biotic stress (Cambier, Hance & De Hoffmann, 2000; Wouters et al., 2016), BX1 to BX14 collectively catalyze the production of multiple glucoside conjugates that can ultimately act as aglycone defenses (Frey, 1997; Jonczyk et al., 2008; Meihls et al., 2013; Handrick et al., 2016). The gene *Bx1* encodes an indole-3-glycerol phosphate lyase that cleaves indole-3-glycerolphosphate into free indole, acting as the first committed step in the pathway (Frey, 1997).

As an example to demonstrate both the power and remaining challenges of using Mutual Ranks to associate pathway genes to one another, we use the reference gene list method to investigate the coexpression of *Bx1* with other *Bx* pathway genes (Fig. 2A; Table S8). Users can select which supporting information to automatically integrate with the MR coexpression table generated (Fig. 2A). The final coexpression table includes columns with the MR values in reference to the first gene in the list (i.e., *Bx1*), gene symbols and gene annotations, and excludes the categories and fold-change columns (Fig. 2A). *Bx6* and *Bx7* were excluded from the coexpression analysis as they were not included in the expression dataset used for this analysis. The coexpression results in the table can be visualized as a coexpression heatmap that readily reveals the highly coexpressed gene cluster of *Bx1* through *Bx5* and *Bx8*, as well as separate coexpression of *Bx10*, *Bx11* and *Bx13* with one another (Fig. 2B). Similar association patterns can also be observed using an interactive coexpression network with an MR < 100 threshold for drawing edges between vertices (Fig. 2C). Using the validated BX pathway as a simplistic MutRank example, we demonstrate the following: (1) the ease of observing strong co-expression of early *Bx* pathway genes, (2) the partial coexpression of late *Bx* pathway genes and (3) remaining challenges of bioinformatically-connecting complex pathways that display differential regulation of early and late steps (Fig. 2D) (Meihls et al., 2013; Handrick et al., 2016). Importantly, biosynthetic pathways function within the complex context of a living cell. The value in confirming established coexpression patterns is to first undertand how the user-defined dataset is performing. When compelling, these results then encourage further interrogation to address diverse hypotheses and complex surrounding processes, potentially identifying coexpressed transcription factors, transporters, or detoxification enzymes to investigate (Lacchini & Goossens, 2020).

### Example workflow 2: using MutRank to predict enzymes in specialized metabolism

**Figure 3:**
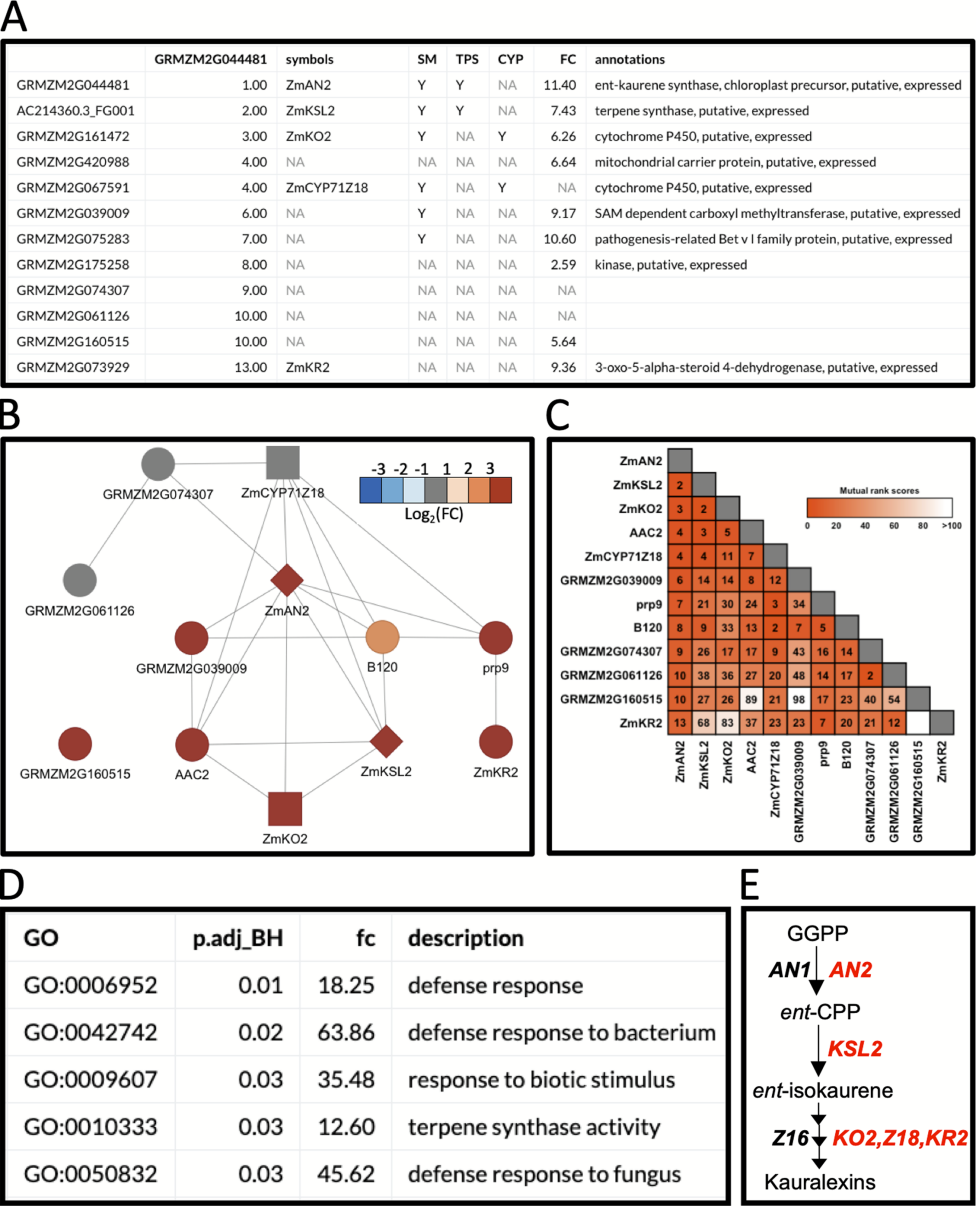
Example workflow 2: using MutRank to predict enzymes in specialized metabolism. (A) Using the kauralexin biosynthetic gene *ANTHER EAR 2* (*AN2*) as a single reference gene, a Mutual Rank (MR)-based coexpression table was generated for the 200 most highly coexpressed genes (first 12 genes are shown) with the integrated supporting information. Using the first 12 genes in the list we generated a (B) coexpression network figure, with an MR < 10 threshold for drawing edges between vertices showing a cluster of coexpressed genes and a (C) coexpression heatmap. (A and B) Genes belonging to a category are denoted with “Y”; the categories used are SM (specialized metabolism), TPS (terpene synthases, diamond shape vertices) and CYP (cytochrome P450s, square shaped vertices). (A and B) Corresponding expression fold change (FC) increase 24 h after pathogen inoculation. (D) Results of the Gene Ontology (GO) term enrichment analysis using the GO-basic database and all genes with MR < 100 are over-represented for terms associated with biotic stress responses. *P*-values were calculated using a hypergeometric test and adjusted using the Bonferroni–Holm method. (E) Summarized diagram of the maize kauralexin biosynthetic pathway showing genes highly coexpressed with the reference gene *AN2* in red.

In the first example, we used BX-related defenses which have been studied in maize and other cereals for over 60 years (Virtanen et al., 1955; Smissman, LaPidus & Beck, 1957). More recently, maize diterpenoid pathways have been implicated in diverse protective roles providing fungal, insect and drought resistance (Schmelz et al., 2011; Vaughan et al., 2015; Christensen et al., 2018; Ding et al., 2019). Biosynthesis of protective *ent*-kaurane-related diterpenoids in maize, termed kauralexins, are mediated by multi-gene terpene synthase (TPS) and cytochrome P450 (CYP) families. Using MR-based coexpression analyses for discovery purposes (Ding et al., 2019) we examined one reference gene termed anther ear 2 (*ZmAN2*) (Table S8), that encodes an *ent*-copalyl diphosphate synthase (*ent*-CPS) responsible for the cyclization of geranylgeranyl diphosphate into bicyclic pathway precursor *ent*-copalyl diphosphate (*ent*-CPP) (Harris et al., 2005). Derived from two different genes encoding *ent*-CPS, *ent*-CPP is a key substrate shared by the kauralexin, dolabralexin and gibberellin biosynthetic pathways in maize (Mafu et al., 2018; Ding et al., 2019). Using *ZmAN2* as a reference gene, we calculated the non-targeted MR values between the top 200 coexpressed genes and integrated the supporting information (Fig. 3A). For simplification, we then selected the first 12 coexpressed genes and identified 1 TPS gene (Figs. 3A and 3B: diamond shaped vertex), a type I diterpene synthase: kaurene synthase-like 2 (*ZmKSL2*) and 2 CYP genes (Figs. 3A and 3B: square shaped vertices), *ZmCYP71Z18* and kaurene oxidase 2 (*ZmKO2*) that were highly coexpressed (Figs. 3A–3C). A GO-term enrichment analysis of the MR-based coexpression results using the GO-basic database revealed an enrichment of terms associated with defense responses and terpene synthesis (Fig. 3D). With candidates identified through similar MR-based coexpression relationships to those currently presented (Figs. 3A–3E), a recent study of kauralexin biosynthetic enzymes were systematically validated using genome wide association studies, heterologous enzyme co-expression assays, proteomics and characterization of defined genetic mutants (Ding et al., 2019). Two additional genes with defined roles in kauralexin biosynthesis that did not match any of the supporting information categories are the *ZmCYP71Z16* that is absent from the currently selected expression dataset and the coexpressed *kauralexin reductase2* (*ZmKR2*) encoding a 5α-steroid reductase that saturates B-series kauralexins (Figs. 3A–3C) (Ding et al., 2019). Together the combined use of MR analyses with biochemistry and defined genetic mutants defined roles for *ZmAn2*, *ZmKSL2*, *ZmKO2, ZmKR2*, *ZmCYP71Z18* and ZmCYP71Z16 in kauralexin biosynthesis and anti-pathogen defense enabling rapid assembly of the entire pathway (Fig. 3E) (Ding et al., 2019). Additional genes identified in the MR-based coexpression analysis encode predicted carrier proteins, pathogenesis-related proteins and kinases that might further contribute to the regulation and transport of diterpenoids (Figs. 3A–3C). In summary, straightforward MR analyses via MutRank provide a powerful starting point for defining networks surrounding specialized metabolism.

## Discussion

MutRank is a user-friendly and powerful tool for exploratory targeted gene coexpression analyses. MutRank enables the simple calculation of MR values for any reference gene or gene set from user-provided expression data. The Shiny web-application interface is ideal for combining MR-based coexpression analyses with useful R packages that produce informative tabular and graphical outputs. We implemented a number of features that allow users to integrate supporting information with the results of the coexpression analyses to facilitate prediction of putative gene functions. Example workflow 1 surveyed genes in the well-established maize BX biosynthetic pathway. Many of these genes were identified and characterized without the benefit of large-scale transcriptomic data (Frey, 1997). The lack of coexpression connections between early and late stage *Bx* biosynthetic genes (Figs. 2A–2D) likely provides a partial explaination for the relatively recent discovery of the terminal steps (Meihls et al., 2013; Handrick et al., 2016).

Public coexpression databases and tools, such as MutRank, provide intuitive user control over MR-based coexpression analyses to drive predictions and hypothesis testing of genes with currently unknown functions. Example workflow 2 was given as an example where MR-based coexpression analyses were used to guide recent hypothesis testing, and through a combination of diverse approaches, were demonstrated to correctly predict gene functions in the maize kauralexin antibiotic pathway (Ding et al., 2019). Importantly, we note here that custom use of further expression datasets were used to correctly predict the function of an additional kauralexin biosynthetic genes (*ZmCYP71Z16*) within the pathway using MR-based coexpression analyses (Ding et al., 2019). In Ding et al. (2019) the expression datasets were derived from the National Center for Biotechnology Information Sequence Read Archive project IDs SRP115041 and SRP011480. This esoteric detail speaks to an essentail point. Different MR coexpression patterns can be found in related datasets depending on sample size, plant growth conditions, genotypes used, tissue types, cell types, developemental age, presence or absence of biotic or aboitic stress and countless other factors important to the questions being examined. Given this, aggregate estimations of gene co-expression available on public websites typically fall short in facilitating elucidation of relationships of interest. Rapid progress requires flexible control over the analyses of precise data subsets or of larger aggregated datasets for cross-comparison. MutRank allows for a large number of different datasets to be selected, and quickly analzyed and assessed. Most commonly, the search for meaningful coexpression relationships, whether of biosynthetic genes or for more complex regulatory processes, is a guided and highly iterative discovery process, relying on partial insights from related experimental systems. A common goal is to generate high-quality gene candidates for improved hypothesis testing that ultimately informs more expensive and time-consuming *in planta* analyses of defined mutants. As a further recent example, MR-based coexpression analyses were leveraged and played a key role in defining and disentangling a challenging 10-gene maize sesquiterpenoid antibiotic pathway partially sharing kauralexin biosynthetic genes (Ding et al., 2020). Research progress in plant specialized metabolism requires rapid, flexible and easy-to-use tools, through which diverse users of varying expertise levels can quickly compare results generated from public or customized user-provided datasets. We now routinely utilize MutRank as a rapid tool for exploratory targeted coexpression analyses facilitating the prediction of functional associations and putative gene functions. The goal of our current effort was to expand the ease and use of the R Shiny web-application tools to facilitate efforts of any biologists who seek to connect coregulated genes to important phenotypes.

## Conclusion

The MutRank R Shiny web application provides an efficient, flexible and simple tool for conducting hypothesis-driven MR-based coexpression analyses. To enable rapid functional discovery, MutRank analyses are integrated with multiple customizable features for narrowing and prioritizing candidate genes and for hypothesis testing in predicted biochemical functions.

## Supplemental Information

10.7717/peerj.10264/supp-1 Supplemental Information 1 Step-by-step guide to reproduce MutRank example workflow results.

10.7717/peerj.10264/supp-2 Supplemental Information 2 MutRank User Manual.

10.7717/peerj.10264/supp-3 Supplemental Information 3 Expression data used to generate MutRank example workflow figures.

10.7717/peerj.10264/supp-4 Supplemental Information 4 Gene annotations, symbols and ontology terms used in MutRank example workflows.

## Additional Information and Declarations

### Data Availability

The following information was supplied regarding data availability: Data are available in the Supplemental Tables and at GitHub. Code is available at GitHub: https://github.com/eporetsky/MutRank.
