# Supplementary material for: MutRank: an R shiny web-application for exploratory targeted mutual rank-based coexpression analyses integrated with user-provided supporting information
Source: PeerJ. 2020 Nov 9;8:e10264. doi: 10.7717/peerj.10264 (PMC7659623; doi:10.7717/peerj.10264)
Supplement: Supplemental Information 1 [file peerj-08-10264-s001.docx]

**Step-by-step Guide to Reproduce the MutRank Example Workflow Results**

**Example workflow 1: Integrating coexpression analyses of genes encoding a specialized metabolic pathway with supporting information**

1. In the side panel of the “Data Input” tab select the example files below:


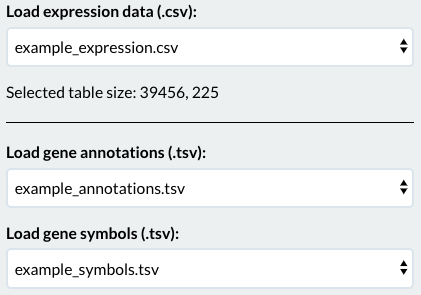


1. Select the “Mutual Rank” tab at the top of the MutRank app
2. Select “Reference gene list” as the reference gene method
3. Paste into the ﻿ “List of reference genes” box the list of Bx genes from Supplementary Table 8 or directly from the gene list below: GRMZM2G085381,GRMZM2G085661,GRMZM2G167549,GRMZM2G172491,GRMZM2G063756,GRMZM2G085054,GRMZM2G161335,GRMZM2G311036,GRMZM2G336824,GRMZM2G023325,AC148152.3_FG005,GRMZM2G127418
4. Press the “Calculate MR Values” button to generate the coexpression table


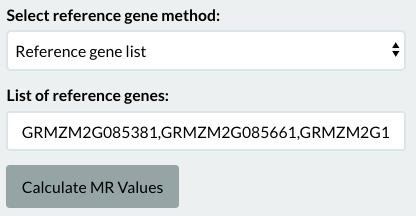


1. Deselect ﻿the “Add custom categories” and “﻿Add foldchange values” options


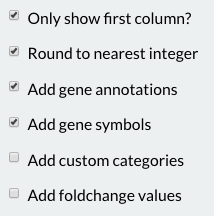


1. **This will reproduce the MR-based coexpression table presented in Figure 2A**
2. Select the “Heatmap” tab at the top of the MutRank app
3. Change the “Text MR threshold” box to 100


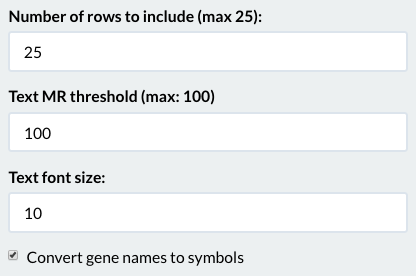


1. **This will reproduce the MR-based coexpression heatmap presented in Figure 2B**
2. Select the “Network” tab at the top of the MutRank app
3. Change the “Select MR Threshold” box to 100
4. Deselect the “Change bait gene to star shape?” and “Color vertices with foldchange data” options


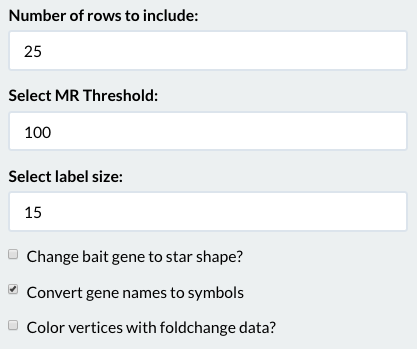


1. **This will reproduce the MR-based coexpression network presented in Figure 2C**

**Example workflow 2: Using MutRank to predict enzymes in specialized metabolism.**

1. In the side panel of the “Data Input” tab select example files below


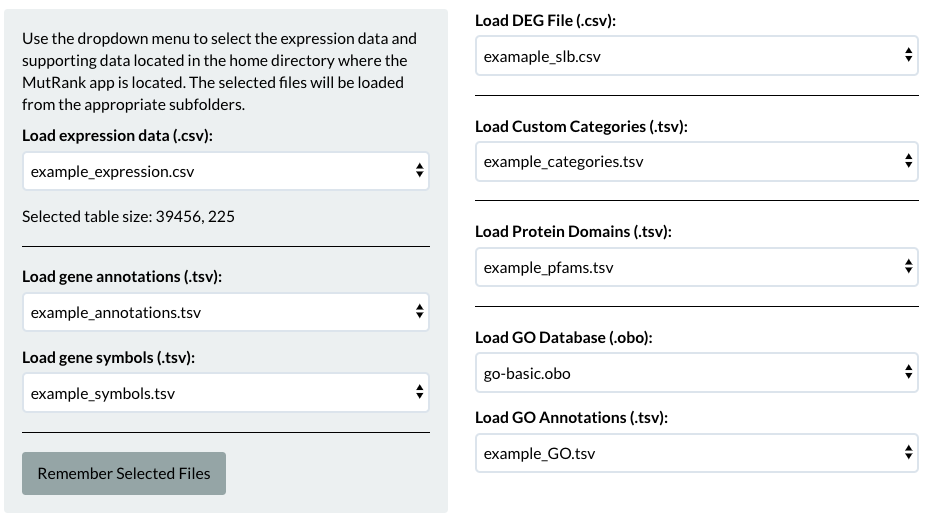


1. Select the “Mutual Rank” tab at the top of the MutRank app
2. Select “Single reference gene” as the reference gene method
3. Paste into the ﻿“List of reference genes” box the gene ID for ZmAN2 from Supplementary Table 8 or use: GRMZM2G044481
4. Press the “Calculate MR Values” button to generate the coexpression table


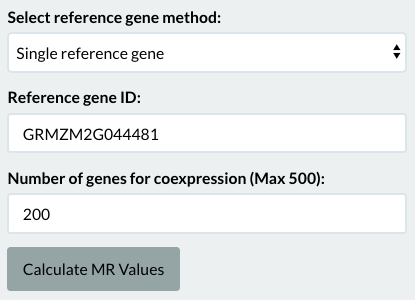


1. Keep all additional options selected


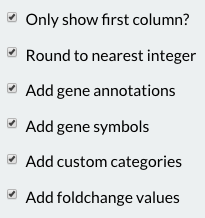


1. **This will reproduce the MR-based coexpression table presented in Figure 3A (Figure 3A is a screenshot of the first 12 genes from this table. The complete table can be found in Supplementary Table 9)**
2. Select the “Network” tab at the top of the MutRank app
3. Change the “Number of rows to include” box to 12
4. Change the “Select MR Threshold” box to 10
5. Deselect the "Change bait gene to star shape?" option and keep all additional options selected


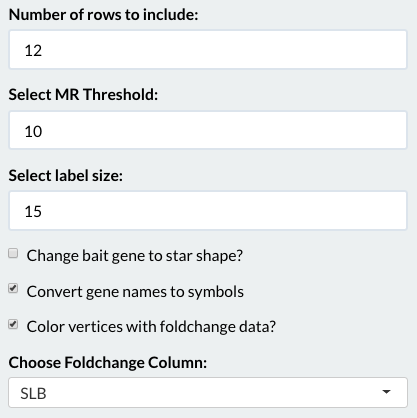


1. In the “Category for diamond shape” select “TPS” and in the “Category for square shape” select “CYP”


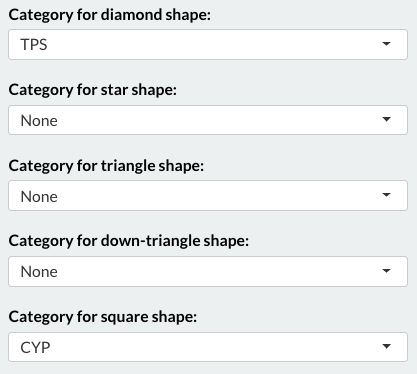


1. **This will reproduce the MR-based coexpression network presented in Figure 3B**
2. Select the “Heatmap” tab at the top of the MutRank app
3. Change the “Number of rows to include” box to 12
4. Change the “Text MR threshold” box to 100


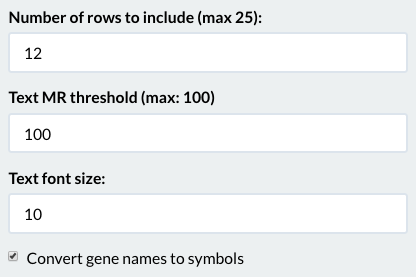


1. **This will reproduce the MR-based coexpression heatmap presented in Figure 3C**
2. Select the “Enrichment” tab at the top of the MutRank app
3. Deselct the “Include non-adjusted p values?” option


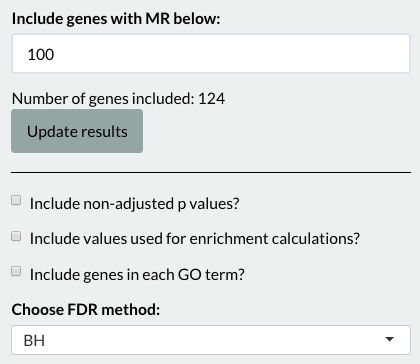


1. **This will reproduce the MR-based GO enrichment table presented in Figure 3D**
