# Supplementary material for: MutRank: an R shiny web-application for exploratory targeted mutual rank-based coexpression analyses integrated with user-provided supporting information
Source: PeerJ. 2020 Nov 9;8:e10264. doi: 10.7717/peerj.10264 (PMC7659623; doi:10.7717/peerj.10264)
Supplement: Supplemental Information 2 [file peerj-08-10264-s002.pdf]

MutRank

Elly Poretsky, Alisa Huffaker

August 24, 2020

Contents

1. Introduction **1**

2. Getting Started **2**
2.1. Requirements 2
2.2. Installation 2
2.3. R Dependencies 2
2.4. Folders and Example Data Sources 2
2.5. File Formats Used 2

3. Guide to Using MutRank **3**
3.1. `Data Input` Tab 3
3.2. `Mutual Rank` Tab 4
3.3. `Heatmap` Tab 5
3.4. `Network` Tab 6
3.5. `Enrichment` Tab 7

4. Credits **8**

5. License **8**

1. Introduction

Transcriptomic data can uncover complex biological processes in part through the improved understanding of gene coexpression patterns. Mutual Rank (MR), the geometric mean of the ranked Pearson's Correlation Coefficient (PCCs) between a pair of genes was shown to be a better indicator of functional associations and produces more robust results when using raw data compared to PCC. It was demonstrated that MR analyses of transcripts should be favored in the prediction pathway gene functions and serve as springboard for hypothesis testing and validation. We developed an R Shiny web-application, termed MutRank, to facilitate user control over both targeted and non-targeted MR-based coexpression analyses for rapid hypothesis testing. In addition to identifying highly coexpressed genes in any user-provided expression dataset, MutRank automatically integrates supporting information such as gene annotations, differential-expression data, predicted domains and assigned GO terms and provides useful tabular and graphical outputs as foundation for empirical hypothesis testing. The goal of MutRank is to provide simple, customizable and readily accessible tools to speed research progress in connecting metabolic phenotypes to genotypes for the purpose of understanding biological roles.

2. Getting Started

2.1. Requirements

- `R` - https://cran.r-project.org/src/base/R-3/
- `R Stduio` - https://rstudio.com/products/rstudio/download/
- `Java` (requires restarting) - https://java.com/en/download/

2.2. Installation

1. Download or clone MutRank from: https://github.com/eporetsky/mutRank
2. Unzip and open the `app.R` file using R Studio
3. To start MutRank press the `Run App` button in R Studio
4. Follow the R instructions to install required R libraries
5. Start using MutRank in the browser or window mode

- MutRank automatically handles installation of required R libraries

2.3. R Dependencies

MutRank will automatically install the packages listed below when when starting the app. The app was tested on windows, linux and macOS with the listed library versions.

- hypergea_1.3.6
- ontologyIndex_2.5
- reshape2_1.4.3
- RColorBrewer_1.1-2
- data.table_1.12.8
- ggplot2_3.3.0
- visNetwork_2.0.9
- igraph_1.2.4.2
- shinythemes_1.1.2
- shiny_1.4.0.2

2.4. Folders and Example Data Sources

- data - Expression data (doi: 10.3835/plantgenome2015.04.0025)
- annotations - Text annotations of genes (https://phytozome.jgi.doe.gov/pz/portal.html)
- symbols - Gene symbols (https://maizegdb.org/gene_center/gene)
- foldchange - Log2 Foldchange data (doi: 10.1038/s41477-019-0509-6)
- categories - Categories assigned to genes, Pfam domains and GO terms (doi: 10.1093/nar/gkw982, doi: 10.1038/s41477-019-0509-6, doi: 10.1105/tpc.17.00009)
- domains - Genes with their predicted domains (https://phytozome.jgi.doe.gov/pz/portal.html)
- GO - GO database (http://geneontology.org/docs/download-ontology/) and assigned terms (https://phytozome.jgi.doe.gov/pz/portal.html)

2.5. File Formats Used

1. Comma-separated values (.csv): Expression and differential expression data
2. Tab-separated values (.tsv) - Annotations, symbols, Pfam domains, GO assignments and custom cetegories
3. Ontology file (.OBO) - Downloaded from http://geneontology.org/docs/download-ontology/

3. Guide to Using MutRank

3.1. Data Input Tab

The Data Input tab is the first tab of the MutRank app in which users can load their expression data and supporting information for MR-based coexpression analyses (Fig. 1). The only requirement to conduct MR-based coexpression analyses is the expression data. Additional user-provided supporting information will be automatically integrated with the coexpression results. When the MutRank app starts, each data folder is parsed to find all files with the expected filename extensions and these files are listed in their relevant dropdown menus. A short delay is expected when loading large expression files, but a short text output containing the table dimensions will update once the expression data file is loaded (Fig. 1A). Non-expressed genes (zero sum expression) are automatically filtered to prevent error messages. User provided supporting information includes gene annotations (Fig. 1B), gene symbols (Fig. 1C), differential expression data (Fig. 1D), custom categories (Fig. 1E), Pfam protein domain annotations (Fig. 1F) and the Gene Ontology (GO) database file along the GO assignments (Fig. 1G). By default MutRank starts with loading the example files but this can be changed by pressing the "Remember Selected Files" button.

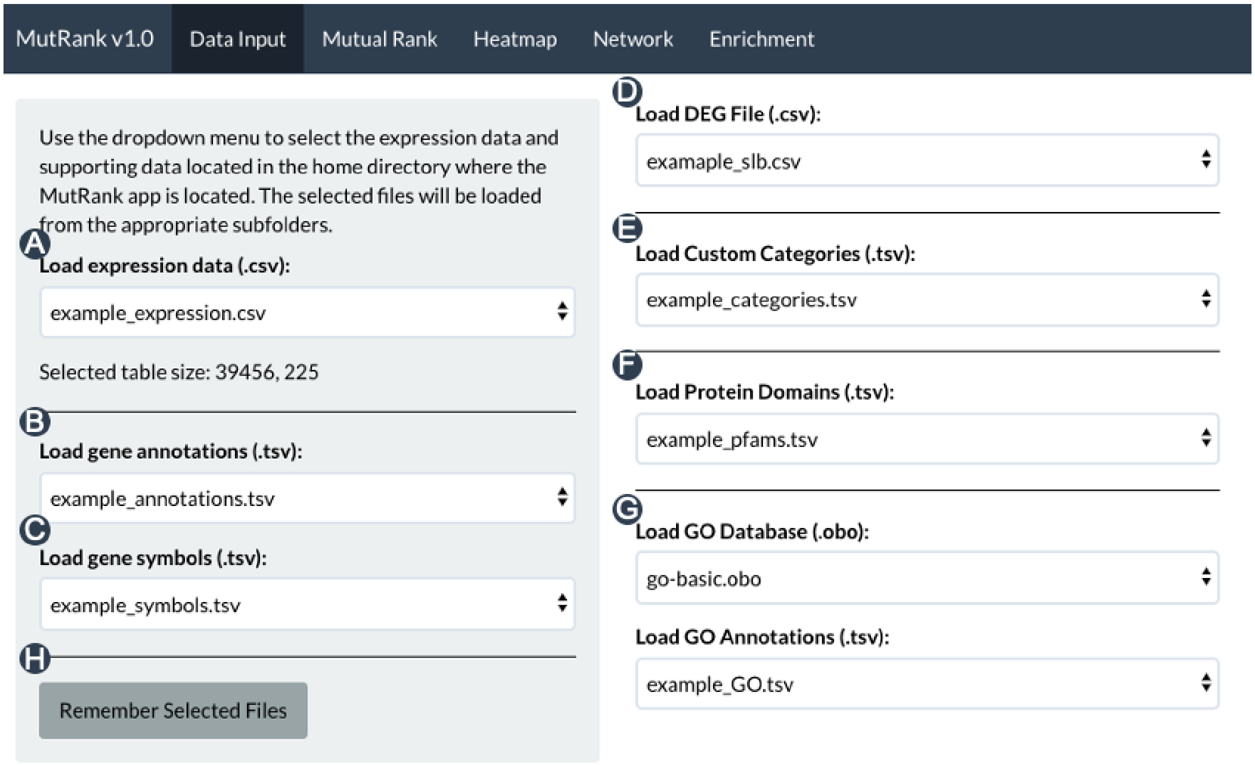

Figure 1: Data Input Tab Screenshot - In the side panel of the users can load the (A) expression data, (B) gene annotations and (C) gene symbols. In the main panel users can load (D) differential expression data, (E) custom categories, (F) protein Pfam domain annotation and (G) the GO database file (notice that in this instance "GO-basic.obo" was used instead of the default "goslim_plant.obo") along the GO assignments. The "Remember Selected Files" button can be used to changed the default files MutRank loads on start (H).

3.2. Mutual Rank Tab

Once the expression data and supporting information are loaded the MR-based coexpression analyses can start (Fig. 2). First, the user should select one of 3 possible reference gene methods: (1) Single reference gene, (2) compound reference gene or (3) reference gene list and then insert a reference or gene list (Fig. 2A). The compound reference gene method creates a new compound reference gene from the calculated average, sum, maximum or minimum expression values of the reference gene list. The reference gene list method calculates the MR values between the genes in the list using the first gene in the list as the primary referenece gene. Gene lists can be separated by: tab, new line, vertical tab, space and comma. By default MutRank will find the 200 coexpressed genes using Pearson's Correlation Coefficient (PCC) values (Fig. 2B) to generate the list of genes for which MR values will be calculated. This practical trade-off between whole genome and targeted coexpression analyses allows MutRank to rapidly complete the analysis and to run on the resources of most personal computers. MR values will be calculated after pressing the "Calculate MR Values" button (Fig. 2C). Additional settings allow the user format the coexpression results and to integrate supporting information (Fig. 2D). The final results will be presented in the MR-based coexpression table in the main panel (Fig. 2E) which can be downloaded as a tsv file using the 'Download Table' button (Fig. 2E).

In the example below (Fig. 2) we selected the maize reference gene GRMZM2G085381 (Bx1).

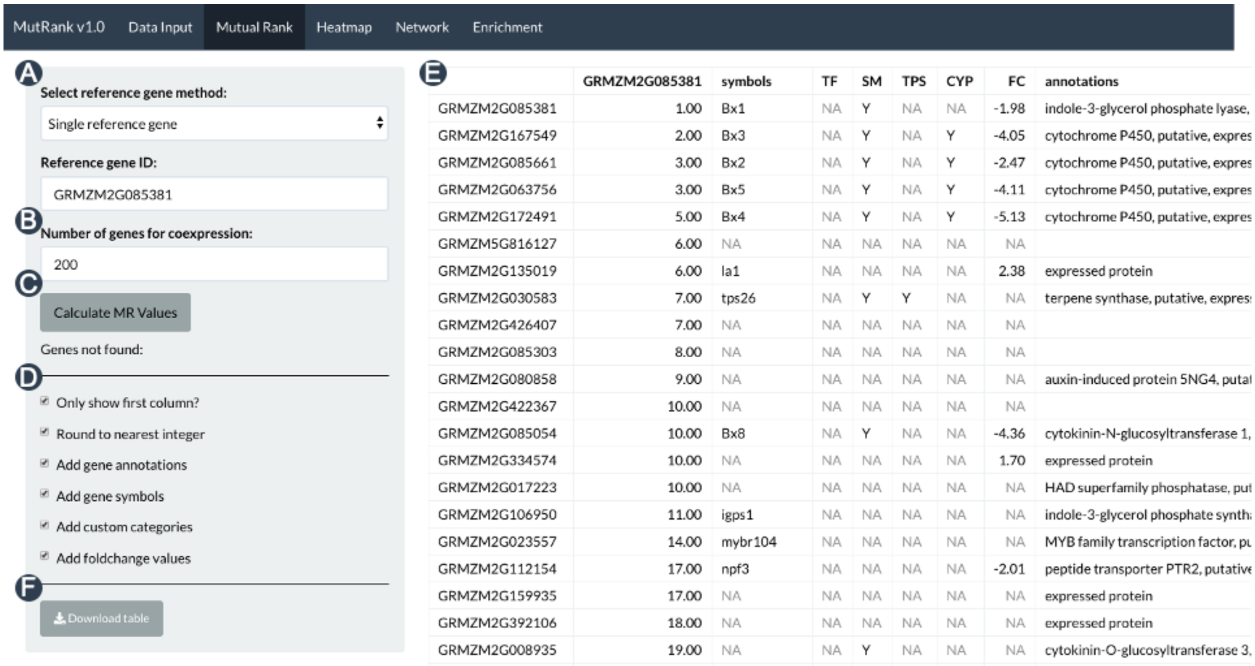

	GRMZM2G085381	symbols	TF	SM	TPS	CYP	FC	annotations
GRMZM2G085381	1.00	Bx1	NA	Y	NA	NA	-1.98	indole-3-glycerol phosphate lyase,
GRMZM2G167549	2.00	Bx3	NA	Y	NA	Y	-4.05	cytochrome P450, putative, expres
GRMZM2G085661	3.00	Bx2	NA	Y	NA	Y	-2.47	cytochrome P450, putative, expres
GRMZM2G063756	3.00	Bx5	NA	Y	NA	Y	-4.11	cytochrome P450, putative, expres
GRMZM2G172491	5.00	Bx4	NA	Y	NA	Y	-5.13	cytochrome P450, putative, expres
GRMZM5G816127	6.00	NA	NA	NA	NA	NA	NA	
GRMZM2G135019	6.00	la1	NA	NA	NA	NA	2.38	expressed protein
GRMZM2G030583	7.00	tps26	NA	Y	Y	NA	NA	terpene synthase, putative, expres
GRMZM2G426407	7.00	NA	NA	NA	NA	NA	NA	
GRMZM2G085303	8.00	NA	NA	NA	NA	NA	NA	
GRMZM2G080858	9.00	NA	NA	NA	NA	NA	NA	auxin-induced protein 5NG4, putat
GRMZM2G422367	10.00	NA	NA	NA	NA	NA	NA	
GRMZM2G085054	10.00	Bx8	NA	Y	NA	NA	-4.36	cytokinin-N-glucosyltransferase 1,
GRMZM2G334574	10.00	NA	NA	NA	NA	NA	1.70	expressed protein
GRMZM2G017223	10.00	NA	NA	NA	NA	NA	NA	HAD superfamily phosphatase, put
GRMZM2G106950	11.00	igps1	NA	NA	NA	NA	NA	indole-3-glycerol phosphate synth
GRMZM2G023557	14.00	mybr104	NA	NA	NA	NA	NA	MYB family transcription factor, pu
GRMZM2G112154	17.00	npf3	NA	NA	NA	NA	-2.01	peptide transporter PTR2, putative
GRMZM2G159935	17.00	NA	NA	NA	NA	NA	NA	expressed protein
GRMZM2G392106	18.00	NA	NA	NA	NA	NA	NA	expressed protein
GRMZM2G008935	19.00	NA	NA	Y	NA	NA	NA	cytokinin-O-glucosyltransferase 3,

Figure 2: Mutual Rank Tab Screenshot - In the side panel users can select (A) the reference gene method and reference gene or gene list and (B) the desired number of the top coexpressed genes (based on PCC values) to include. Pressing the 'Calculate MR Values' button will start the coexpression analysis (C). Users can select additional settings allow will format the coexpression results and integrate supporting information (D). The final MR-based coexpression results will be presented in the table in the main panel (E) which can be downloaded by pressing the 'Download Table' button (F).

3.3. Heatmap Tab

The MR-based coexpression table generated in the Mutual Rank tab can be used to generate a heatmap graphical output in the Heat Map tab (Fig. 3). We set the maximum number of genes to be presented using the heatmap at 25 to keep it intelligible. We have included a few options that allow users to modify the heatmap figure, including the number of genes to included in the heatmap (Fig. 3A), the maximum MR value to be included as text within the heatmap (Fig. 3B), the text size (Fig. 3C) and an option to convert gene IDs to gene symbols, when applicable (Fig. 3D). The red-to-white color gradient is used to represent the MR values and is set to represent MR values between 1 and 100 with all values higher than 100 set to a white color. The heatmap presented in the main panel (Fig. 3E) can be downloaded as a PNG file using the 'Download Heatmap' button (Fig. 3F).

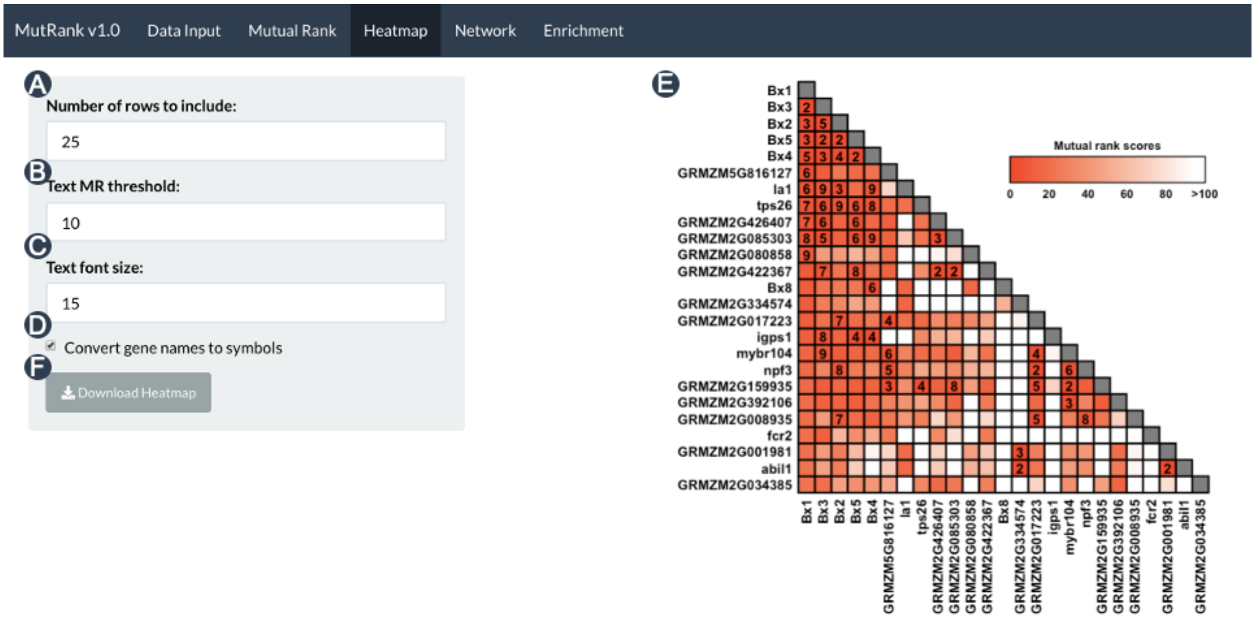

Figure 3: Heatmap Tab Screenshot - In the side panel users can select (A) the number of genes to included in the heatmap, (B) the maximum MR value to be included as text within the heatmap, (C) the text size and (D) an option to convert gene IDs to gene symbols. The heatmap presented in the main panel (E) can be downloaded as a PNG file using the 'Download Heatmap' button (F).

3.4. Network Tab

The MR-based coexpression table generated in the Mutual Rank tab can be used to generate a coexpression network (Fig. 4). First, the MR-based coexpression table is converted to an adjacency matrix using igraph which is used to annotate the gene nodes with the supporting information. Users can select how many of the top coexpressed genes to include in the network (Fig. 4A), the MR threshold to connect to gene vertices with an edge (Fig. 4B), the size of the text labels (Fig. 4C), wether to convert the shape of reference gene vertex to a star (Fig. 4D) and wether to convert the gene IDs of each node to gene symbols, when applicable (Fig. 4E). Differential expression Log2 fold-change values can be integrated by selecting one of the columns from the data to change the color of the gene nodes (Fig. 4F). Custom categories can be integrated by changing the shape of the gene nodes to one of 5 shapes: (1) diamond, (2) start, (3) triangle, (4) down-triangle or (5) square (Fig. 4G). If a gene belongs to more than one custom category it will take the shape of the last shape it belongs to from the previous list. When available, gene annotations are automatically integrated. After assigning all these attributes to the nodes using igraph, the igraph network is converted to a dynamic java-script-based netowrk visualization using the vizNetwork package (Fig. 4H). Gene annotations can accessed by pressing on any of the gene nodes to trigger a pop-up text message (Fig. 4I). Differential expression data is visualized as an incremental gradient between log2 fold-change of -3 and 3. The gradient scale (Fig. 4J) was added manually and is available as a separate image in the “img” folder under the “DEG_gradient.png” file name.

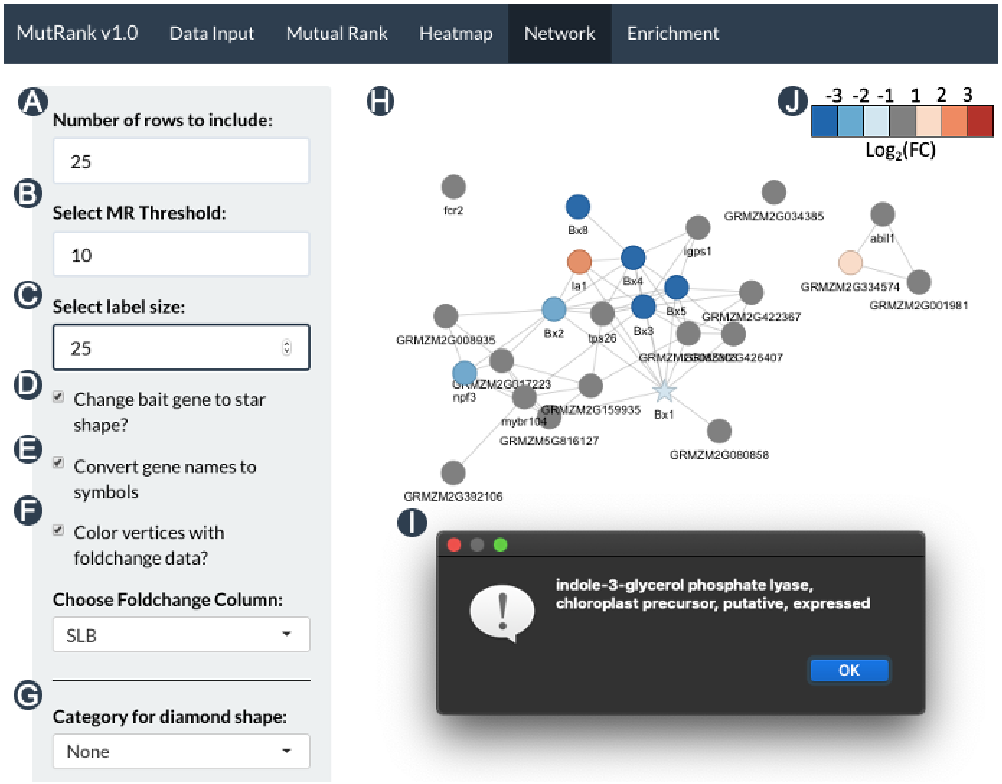

Figure 4: Network Tab Screenshot - In the side panel users can select (A) how many of the top coexpressed genes to include in the network (A), the MR threshold to connect gene vertices with an edge (B), the size of the text labels (C), wether to convert the shape of reference gene vertex to a star (D) and wether to convert the gene IDs of each node to gene symbols, when applicable (E). Differential expression Log2 fold-change values can be integrated by selecting one of the columns from the data to change the color of the gene nodes (F). Custom categories can be integrated by changing the vertex shape (G). In the MR-based coexpression network (H), gene annotations can accessed by pressing on any of the gene nodes to trigger a pop-up text message (4I). The gradient scale (J) was added manually after the screenshot was taken.

3.5. Enrichment Tab

The MR-based coexpression table generated in the Mutual Rank tab can be tested for Gene Ontology (GO) enrichment. We use the hypergeometric test using the selected GO database to calculate the P-values for GO term enrichment (Fig. 5). In the side panel users can select MR threshold that will be used to include genes for the enrichment analysis (Fig. 4A). Users can also choose to include in the final table, for each GO term, the non-adjusted p-values, the values used for the hypergeometric test and the list of genes included in the analysis (Fig. 5B). The column names used for the values used for the hypergeometric test are: “N” - Number of genes in the GO annotation files; “M” - Number of genes annotated with specific GO term; “n” - Number of included genes from the coexpression table; “m” - Number of included genes from the coexpression table that are annotated with the specific GO term. Users can also select which method (holm, hochberg, hommel, bonferroni, BH or BY) to use to adjust the P-value for false-discvery rate (FDR) (Fig. 5C). The GO enrichment table presented in the main panel (Fig. 5D) can be downloaded as a PNG file using the ‘Download Table’ button (Fig. 3E).

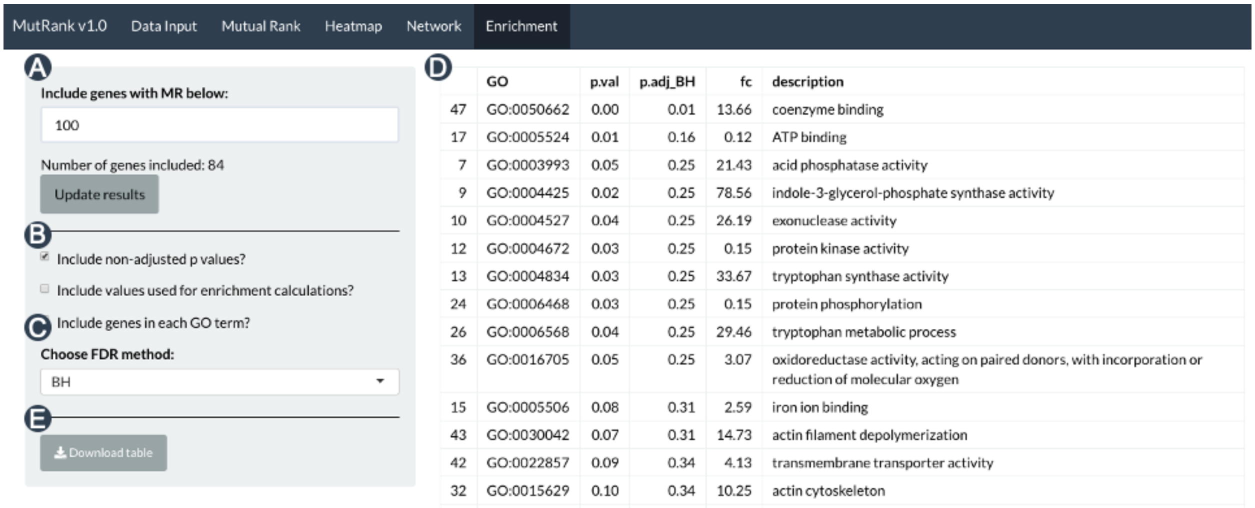

Figure 5: Enrichment Tab Screenshot - In the side panel users can select MR threshold that will be used to include genes for the enrichment analysis (A). Users can choose to include in the final table, for each GO term, the non-adjusted p-values, the values used for the hypergeometric test and the list of genes included in the analysis (B). Users also select which method to use to adjust the P-value for false-discvery rate (FDR) (C). The GO enrichment table presented in the main panel (D) can be downloaded as a PNG file using the ‘Download Table’ button (E).

4. Credits

MutRank was conceived by Elly Poretsky and Alisa Huffaker and implemented by Elly Poretsky. We are grateful to Eric A. Schmelz for providing helpful comments in process of developing MutRank and on the corresponding manuscript. The MutRank web-application was made possible with R, R Studio, Shiny and the additional dependencies mentioned in this manual.

5. License

MutRank is available under the terms of the Creative Commons Attribution License (CC BY-NC 3.0).
